# The “Second Hit” of Repair in a Rabbit Model of Chronic Rotator Cuff Tear

**DOI:** 10.3389/fphys.2022.801829

**Published:** 2022-03-08

**Authors:** Isabella T. Wu, Michael C. Gibbons, Mary C. Esparza, Laura S. Vasquez-Bolanos, Sydnee A. Hyman, Shanelle N. Dorn, Anshuman Singh, John G. Lane, Donald C. Fithian, Severin Ruoss, Samuel R. Ward

**Affiliations:** ^1^Department of Orthopaedic Surgery, University of California, San Diego, San Diego, CA, United States; ^2^Department of Bioengineering, University of California, San Diego, San Diego, CA, United States; ^3^Department of Orthopaedic Surgery, Kaiser Permanente, San Diego, CA, United States; ^4^Department of Radiology, University of California, San Diego, San Diego, CA, United States

**Keywords:** rotator cuff, shoulder, animal model, muscle physiology, muscle degeneration

## Abstract

The rabbit supraspinatus is a useful translational model for rotator cuff (RC) repair because it recapitulates muscle atrophy and fat accumulation observed in humans after a chronic tear (the “first hit”). However, a timeline of RC tissue response after repair, especially with regard to recent evidence of muscle degeneration and lack of regeneration, is currently unavailable. Thus, the purpose of this study was to characterize the progression of muscle and fat changes over time after the repair of a chronic RC tear in the rabbit model. Two rounds of experiments were conducted in 2017–2018 and 2019–2020 with *N* = 18 and 16 skeletally mature New Zealand White rabbits, respectively. Animals underwent left supraspinatus tenotomy with repair 8 weeks later. The unoperated right shoulder served as control. The rabbits were sacrificed at 1-, 2-, 4-, and 8-weeks post-repair for histological and biochemical analysis. Atrophy, measured by fiber cross-sectional area and muscle mass, was greatest around 2 weeks after repair. Active muscle degeneration peaked at the same time, involving 8% of slide areas. There was no significant regeneration at any timepoint. Fat accumulation and fibrosis were significantly increased across all time points compared to contralateral. Statement of Clinical Significance: These results demonstrate model reproducibility and a “second hit” phenomenon of repair-induced muscle atrophy and degeneration which partially recovers after a short time, while increased fat and fibrosis persist.

## Introduction

Rotator cuff (RC) repair is one of the most common orthopedic procedures, yet it suffers from a non-healing or retear rate of roughly 25% for chronic small-to-medium tears, (Cho and Rhee, 2009; McElvany et al., 2015; Kwon et al., 2019) and up to 90% in large or massive tears (Galatz et al., 2004). Studies have associated repair failure with a number of factors, including chronicity, patient characteristics, surgical technique, and/or the nature of the RC tear itself (Cho and Rhee, 2009; McElvany et al., 2015; Park et al., 2015; Kwon et al., 2019). Of these, tear size, retraction, and fatty infiltration are some of the most consistently observed risk factors for retear (Cho and Rhee, 2009; McElvany et al., 2015; Park et al., 2015; Kwon et al., 2019). These tissue changes after tear can be considered the “first hit” to the muscle and tendon. It has been shown that even successful repairs do not reverse muscle atrophy and fat accumulation, nor prevent disease progression (Gladstone et al., 2007; Cho and Rhee, 2009; Kwon et al., 2019). However, limited data are available to determine if RC repair surgery generates a similar, secondary injury – a “second hit.”

Rotator cuff tears and repairs have been simulated in a wide range of animal models, each with strengths and weaknesses. Large animal models, such as sheep, generate an injury phenotype with the greatest similarity to human disease (Gerber et al., 2004), but suffer from challenges in cost, complexity of management (Derwin et al., 2010), and limited biological tools. Small animals, such as mice and rats, are more accessible but do not replicate the muscle phenotype seen in human RC tears unless combined with denervation or other interventions unspecific to the typical chronic RC tear patient (Barton et al., 2005; Liu et al., 2011, 2012). Nerve injuries in particular have been associated with a relatively small percentage of human tears (Collin et al., 2014; Shi et al., 2014). The rabbit has emerged as a middle ground model for studying the mechanism and treatment of RC muscle pathology, because they do not require nerve injury to demonstrate fat accumulation after supraspinatus tenotomy, and do not suffer from the higher costs or management complexity (Fabis et al., 1998, 2000; Matsumoto et al., 2002; Uhthoff et al., 2003; Trudel et al., 2019). Furthermore, rabbit repair models have been utilized to study different RC surgical techniques (Ozbaydar et al., 2008; Li et al., 2018; Su et al., 2018; Sun et al., 2020), augmentation strategies (Chung et al., 2013), and biologic therapies (Honda et al., 2017; Kwon et al., 2018; Yoon et al., 2018), in the hopes of improving retear rates and/or reversing fat accumulation and atrophy.

Despite this, no studies to our knowledge have established a time-specific baseline of the RC tissue response after repair in these animals. This is essential to further investigate whether a “second hit” to the tissue structure occurs after repair surgery. Earlier studies did show muscle atrophy and fatty infiltration after a chronic tear (Fabis et al., 1998, 2000; Valencia et al., 2018), which was not reversed by repair (Matsumoto et al., 2002; Uhthoff et al., 2003). However, recent work has provided additional evidence of active muscle degeneration, with absent or minimal regeneration, after rabbit supraspinatus tenotomy (Vargas-Vila et al., 2021). These findings have yet to be investigated in the context of repair. Furthermore, this new data provides a detailed exploration of the relationship between muscle degeneration and adjacent fat accumulation at the muscle fascicle level (Vargas-Vila et al., 2021). Again, the effect of repair on these changes has been unknown. Finally, previous studies focused primarily on the timing of repair (Matsumoto et al., 2002; Uhthoff et al., 2003, 2014a) not the timing of changes afterward. Examining the reproducibility of this animal model between different surgeons and studies would potentially provide more certainty that the observed differences between studies are due to repair timing alone.

Thus, the purpose of this study was to characterize the progression of muscle and fat changes over time after the repair of a chronic RC tear in a rabbit model. The hypotheses were: (a) an acute worsening of muscle atrophy will occur at 1–2 weeks post-repair, (b) followed by a lack of regeneration and (c) later, stable or gradual progression of muscle atrophy, degeneration, and fatty infiltration. A secondary purpose was to investigate the robustness of this rabbit chronic RC tear model through a second series of experiments performed by different surgeons.

## Materials and Methods

### Animals

All protocols were approved by the UC San Diego Institutional Animal Care and Use Committee (protocol # S11246) prior to commencement of the study. The animal subjects were skeletally mature (6-month-old) female New Zealand White rabbits (*oryctolagus cuniculus*). All rabbits were planned to undergo surgical tenotomy of the left supraspinatus, followed by RC repair 8 weeks later. The right shoulder remained unoperated. Then the animals were sacrificed at 1, 2, 4, or 8 weeks after repair.

During both rounds of experimentation, rabbits were single housed in cages. They were provided food and water ad lib, environmental and food enrichment, and visual access to other animals. Assigned identification numbers were randomized using Microsoft Excel to the timepoints. Researchers were aware of the group allocation during the surgeries and tissue harvest. Specimens were labeled with ID numbers only. Criteria for humane endpoints established at the beginning of the study included: displaying clinical signs of disease, loss of appetite, weight below 15% of what is expected for the animal, and/or signs of distress, such as self-mutilation.

The first round of experiments was conducted in 2017–2018 (hereafter referred to as the “2018” group) with 18 rabbits. There were initially four rabbits in the 1- and 8-week post-repair groups, and five rabbits in the 2- and 4-week groups. One rabbit in the 4-week group was sacrificed before repair due to lack of appetite and weight loss, leaving *N* = 4 at every timepoint except 2 weeks (*N* = 5).

A second round of identical experiments was performed in 2019–2020 (hereafter referred to as the “2020” group) with another 16 rabbits (*N* = 4 per group). In 2020, one rabbit in the 2-week group chewed through the endotracheal tube in recovery and underwent humane euthanasia, leaving *N* = 4 at every timepoint except 2 weeks (*N* = 3).

### Surgical Procedures

The surgeries were performed by different authors in the different rounds of experimentation. In 2018, MG, AS, and DF performed the tenotomies while MG, JL, and DF did the repairs. In 2020, DF and AS performed the tenotomies and JL and SH performed the repairs. Rabbits underwent general anesthesia induction using a subcutaneous injection of ketamine and xylazine, followed by intubation. Anesthesia was maintained using 2–4% isoflurane vapor. The left supraspinatus muscle served as the experimental side in all animals, with the right shoulder as an unoperated control, as described previously (Vargas-Vila et al., 2021). In brief, an open anterior approach was performed on the left shoulder, followed by sharp transection of the left supraspinatus tendon from its footprint on the greater tuberosity of the humerus. The surrounding soft tissues were bluntly dissected to allow unhindered retraction of the tendon stump and distal muscle. After securing a Penrose drain to the tendon stump to prevent scar formation between the tendon and surrounding soft tissue, the incision was closed in layers.

Rabbits were then allowed individual cage activity with routine post-operative care. A fentanyl patch was placed on the back for pain control for 3 days, and the animals were monitored daily for 2 weeks post-operatively. At 8 weeks post-tenotomy, all animals underwent an open repair of the torn tendon. The repair was performed using a modified locking suture with anterior and posterior bone tunnels to restore the tendon footprint to the humeral head. The anesthesia, surgical approach and closure, and post-operative protocols were the same as above. Finally, four rabbits were euthanized at each timepoint (1-, 2-, 4-, and 8-weeks post-repair). Repair integrity was assessed by visual inspection, and any re-tears or unusual findings were recorded and reported. The bilateral shoulders were resected en bloc, and the bilateral supraspinatus muscles were harvested from each fossa.

### Tissue Analysis

The mass of each supraspinatus muscle was recorded to the nearest hundredth of a gram (Ohaus Ranger digital scale). The muscle was subdivided into four regions: anterior lateral (A1), anterior medial (A2), posterior lateral (P1), and posterior medial (P2), with the central tendon dividing the anterior versus posterior regions. These sections were pinned at *in vivo* length, snap-frozen in liquid nitrogen-cooled isopentane, and then stored at -80^°^C. After being embedded in OCT, the frozen muscle was sectioned in a cryostat to generate axial and longitudinal sections.

Muscle atrophy, degeneration, and regeneration were quantified using histology. Muscle fiber cross-sectional area (CSA) was used as a marker of atrophy, while the percentage of centrally nucleated fibers (% CN) was used to represent regeneration. Sections were stained with wheat germ agglutinin (WGA) and 4’,6-diamidino-2-phenylindole (Vector Vectashield with DAPI). CSA and % CN were then measured with a custom ImageJ macro. Muscle degeneration was evaluated on hematoxylin and eosin-stained (H&E) slides with an overlaid 500 μm^2^ grid. Each grid section was marked positive or negative for signs of muscle fiber degeneration, as described previous (Gibbons et al., 2017; Vargas-Vila et al., 2021).

Myosin heavy chain (MHC) isoform composition was analyzed using sodium dodecyl sulfate-polyacrylamide gel electrophoresis (SDS-PAGE), similar to previous studies (Talmadge and Roy, 1993; Vargas-Vila et al., 2021). In brief, samples were prepared from muscle sections and loaded into lanes on the gel. The control lanes were loaded with homogenized adult rat soleus muscle standard containing type I, IIa, IIb, and IIx MHC isoforms. After the gels were run and silver-stained, MHC sample bands were compared to the rat standard for identification. The percent composition of each MHC isoform was calculated according to the relative intensity of each band (GS-800, BioRad).

Fatty infiltration was assessed as the percentage of total CSA which stained red for lipid on Oil Red O (ORO) staining (Phillips et al., 1996) using a semi-automated thresholding protocol in Metamorph. A secondary method of fat quantification was performed using the H&E-stained slides and grading each grid element positive or negative for lipid deposits. Fibrosis was also analyzed using two separate methods: histology and bulk hydroxyproline assay. Slides underwent trichrome staining (PolyScience Masson’s Trichrome Kit) followed by Metamorph manual threshold analysis to determine the percentage of total CSA that stained blue for collagen. The bulk hydroxyproline assay was performed using established methods (Edwards and O’Brien, 1980; Zhou and Moore, 2017) and then hydroxyproline content was converted to collagen content (Rowshan et al., 2010), similar to previous studies (Vargas-Vila et al., 2021).

### Statistical Analysis

The first set of analyses was conducted to determine if there were differences between the two rounds of experimentation (2018 and 2020) at each timepoint (1-, 2-, 4-, or 8-weeks post-repair) within each group (repair or control). Data were compared for each timepoint and supraspinatus muscle region (A1, A2, P1, and P2) between years using unpaired *t*-tests. Each dependent variable for each rabbit was then averaged across all muscle regions and compared at each timepoint between years using unpaired *t*-tests (see Supplementary Figures). This analytical approach maximized the probability of identifying differences between cohort of animals. Finally, one-way ANOVAs were used to compare the differences between 2018 and 2020 for each variable (muscle mass, fiber CSA, % degeneration, % central nuclei, % fat on ORO, % fat on H&E, collagen content using OHP, collagen content on trichrome staining).

**FIGURE 1:**
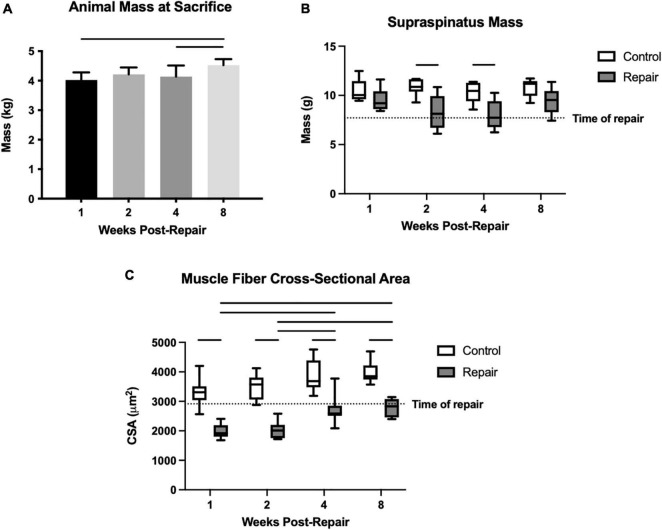
Overall animal mass increased significantly over time **(A)**. Mean supraspinatus mass remained stable on the control side, but the repaired side decreased significantly in mass compared to control at 2 and 4 weeks post-repair **(B)**. Muscle fiber cross-sectional area (CSA) was decreased compared to control at every timepoint, but increased from 1–2 weeks to 4–8 weeks, while the control fiber CSA remained similar throughout **(C)**. Data presented as mean ± SD for bar graph, or minimum and maximum for box plot, *N* = 8 per timepoint. Significant comparisons (*p* < 0.05) within timepoint or within treatment group are indicated by horizontal line. Dotted line represents historical control data from previous study (Vargas-Vila et al., 2021) at 8 weeks after tenotomy (time of repair).

**FIGURE 2:**
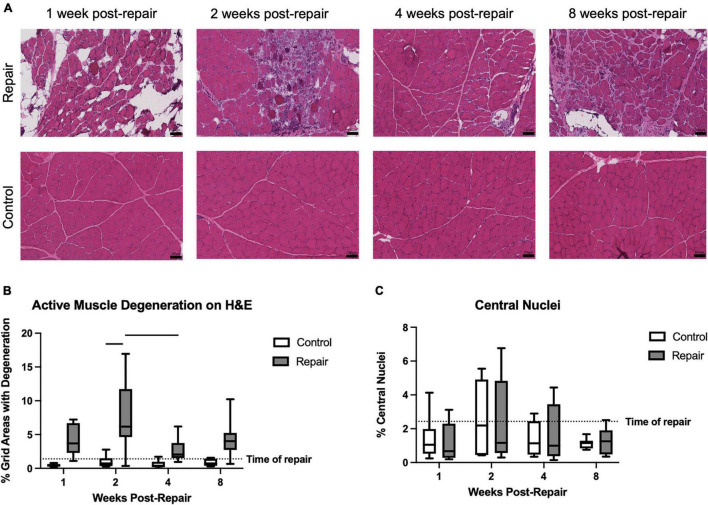
Representative H&E images of repaired and unoperated control supraspinatus muscle at various timepoints **(A)**. Significant active muscle degeneration was seen at 2 weeks post-repair, compared to contralateral, which decreased at 4 weeks **(B)**. No significant regeneration, represented by percentage of centralized nuclei, was observed **(C)**. Data presented as mean ± SD for bar graph, or minimum and maximum for box plot, *N* = 8 per timepoint. Significant comparisons (*p* < 0.05) within timepoint or within treatment group are indicated by horizontal line. Dotted line represents historical control data from previous study (Vargas-Vila et al., 2021) at 8 weeks after tenotomy (time of repair).

**FIGURE 3:**
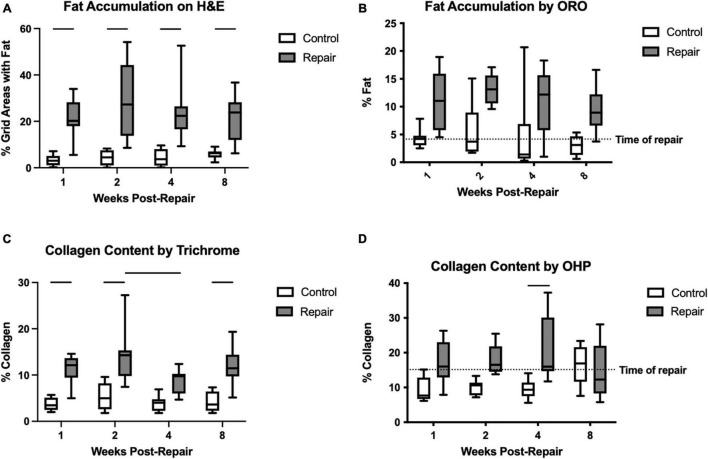
Fat accumulation was significantly increased on the repair side compared to control for every timepoint on H&E staining **(A)**. Similarly, for ORO, the main effect of treatment was significant but pairwise comparisons were not **(B)**. Collagen content as a marker of fibrosis was increased after repair as well on trichrome staining **(C)** and hydroxyproline assay **(D)**. Data presented as mean ± SD for bar graph, or minimum and maximum for box plot, *N* = 8 per timepoint. Significant comparisons (*p* < 0.05) within timepoint or within treatment group are indicated by horizontal line. Dotted line represents historical control data from previous study (Vargas-Vila et al., 2021) at 8 weeks after tenotomy (time of repair).

**FIGURE 4:**
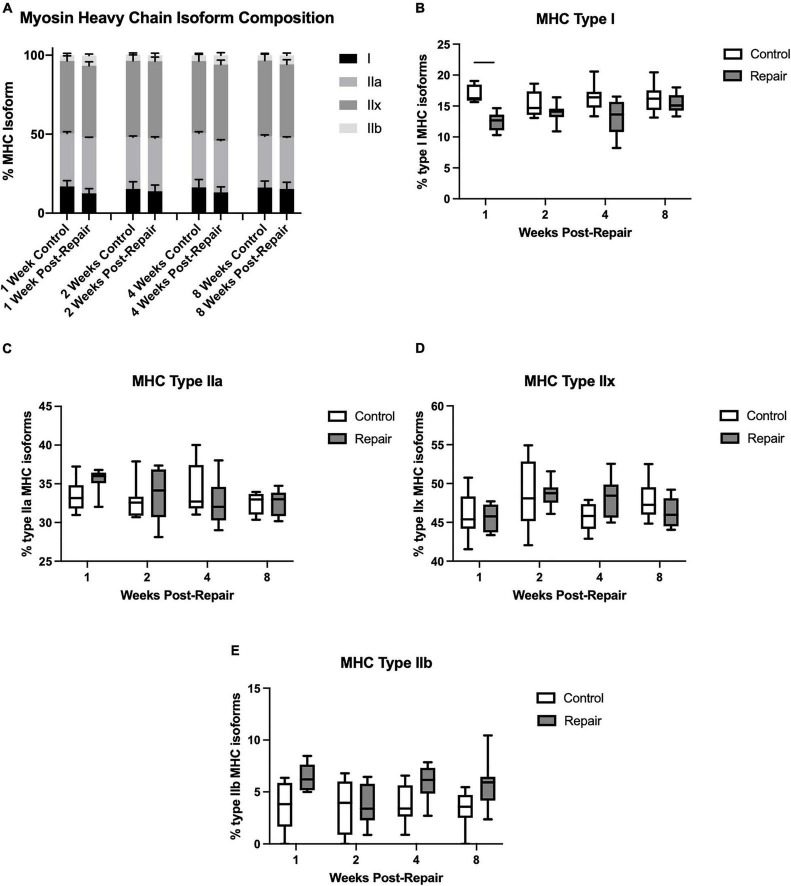
Myosin heavy chain isoform averaged across all regions **(A)**. Percentage of MHC type I decreased at 1-week post-repair **(B)**, but type IIa **(C)**, IIx **(D)**, and IIb **(E)** remained unchanged. Data presented as mean ± SEM for bar graph, or minimum and maximum for box plot, *N* = 8 per timepoint. Significant comparisons (*p* < 0.05) within timepoint or within treatment group are indicated by horizontal line. Dotted line represents historical control data from previous study (Vargas-Vila et al., 2021) at 8 weeks after tenotomy (time of repair).

The second set of analyses was conducted to determine if there were differences between the repair and control groups at each timepoint. One-way ANOVA was used for the rabbit body mass over time. Whole muscle data were again calculated as the mean of measurements across all regions of the same muscle, and two-way ANOVAs were used to compare different independent variables between sides over time. All analyses were conducted using GraphPad Prism (version 8; San Diego, CA, United States) with a level of significance set to α = 0.05 for ANOVAs, and family wise for *post hoc* pairwise comparisons. To qualitatively compare changes from the time of repair, the mean 8-week post-tenotomy data (*n* = 6) from a similar prior study (Vargas-Vila et al., 2021) are illustrated in Figures 1–4. Statistical comparisons were not performed between the current study and the previous one, as a sham surgery was performed on the control side in the historical cohort.

## Results

At the time of euthanasia, all operated shoulders were examined for evidence of re-tear or unusual findings. No re-tears occurred in the 2018 groups. One rabbit in the 2-week group suffered a clavicle fracture, swelling, and a local hematoma after surgery. In the 2020 cohort, there was one with medial migration of the sutures and some qualitative appearance of retraction. However, neither of these animals were outliers in any dependent variable, so they were retained in the analysis. An additional three rabbits showed evidence of a firm nodule close to or at the repair site which seemed fibrocartilaginous in nature, but again these observations did not relate to dependent variable changes. The univariate ANOVAs showed that, of all variables tested, only the percentage of centralized nuclei was significantly different between groups (0.7 ± 0.4% in 2018 vs. 2.4 ± 1.7% in 2020) (*p* < 0.001). Therefore, the 2018 and 2020 cohorts were combined.

### Pooled Experimental Data

Though animals were skeletally mature at the beginning of the study, their body masses did grow significantly by 12.6 ± 0.03% from week 1 to week 8 (overall effect, *p* = 0.0058) (Figure 1). Supraspinatus muscle mass at 1 week after repair was statistically similar to the contralateral shoulder. Then it decreased to a 26 ± 6% and 26 ± 7% difference (repair vs. control) at 2 and 4 weeks respectively, before returning to a similar mass (effect of treatment, *p* < 0.0001) (Figure 1). The post-repair shoulders showed persistent muscle atrophy, as quantified by the muscle fiber CSA, at every timepoint compared to the control side (all *p* < 0.05) (Figure 1). Although muscle fiber CSA increased on the repair side from 2 weeks to 4 weeks, the absolute size of muscle fibers remained lower than the control side. There were no significant changes between timepoints on the control side (effect of treatment, *p* < 0.0001; effect of time, *p* = 0.0357; no interaction). The percentage difference in fiber CSA between repair and control muscles was greatest at 2 weeks post-repair (54 ± 7%) followed by 1-week post-repair (51 ± 8%) but recovered to 35 ± 6% and 35 ± 6% at 4 and 8 weeks, respectively.

Signs of active muscle degeneration significantly increased at 2 weeks post-repair, involving approximately 8 ± 5% of the areas delineated by an overlaid grid (Figure 2). This increase in degeneration decreased by 4 weeks to a level similar to the control side (effect of time, *p* = 0.0190; no interaction). Mean degeneration on the control side did not increase above 1% at any timepoint (overall effect of treatment, *p* < 0.0001). The percentage of centralized nuclei, as a marker of muscle regeneration, did not change after repair or over time (Figure 2).

Fat accumulation on H&E staining increased significantly after repair at all timepoints compared to the contralateral side (Figure 3). Similarly, using ORO staining, the repaired muscle demonstrated significant elevations in fat content (main effect of treatment *p* < 0.0001), but the pair-wise within-timepoint comparisons did not reach significance (Figure 3). Fibrosis was increased significantly in the repair versus control muscles using trichrome (effect of treatment, *p* < 0.0001; effect of time, *p* = 0.0381; no interaction) or OHP (effect of treatment, *p* < 0.0001; effect of time, *p* = n.s.; interaction, *p* = 0.0241). Pairwise comparisons within each technique suggested significant increases at 1, 2, and 8 weeks using trichrome, and at 4 weeks using OHP (Figure 3).

Myosin heavy chain isoform composition showed limited changes after repair (Figure 4). The percentage of MHC type I decreased in week 1 on the repair side compared to control (*p* = 0.0012), but otherwise there were no significant differences. The main effect of treatment was significant for type I (*p* < 0.0001) and type IIb (*p* = 0.0003), while type IIx showed a significant effect of time (*p* = 0.0308).

### 2018 vs. 2020 Experiments

The results from the two rounds of experimentation (2018 versus 2020) were analyzed for differences, comparing the same timepoint (1-, 2-, 4-, or 8-weeks post-repair) and experimental condition (right-sided control, left-sided repair). There were no differences in the average mass of the rabbits at each timepoint (Supplementary Figure 1). The mean mass of the supraspinatus muscle at time of sacrifice showed a significant difference between cohorts only at 1-week post-repair (Supplementary Figure 2). Muscle fiber CSA, another marker of muscle atrophy, also demonstrated no differences between study years (Supplementary Figure 2).

Signs of muscle degeneration were present in a similar percentage of grid areas with the exception of 1-week post-repair animals (Supplementary Figure 3). Muscle regeneration, quantified as the percentage of muscle fibers with centralized nuclei, was significantly higher in the 2018 study for the 2- and 4-week control and the 4-week repair animals, but remained below 4% in all measurements (Supplementary Figure 3).

Fatty infiltration quantified with ORO staining showed no significant differences at any timepoint between study years (Supplementary Figure 4). Fibrosis was measured using two methods of quantifying collagen content: hydroxyproline assay and trichrome staining. Both demonstrated control-side differences only (at week 2 for hydroxyproline assay and weeks 1, 2, and 8 for trichrome staining; Supplementary Figure 5).

Power analysis was performed to determine minimum sample size requirements for future repair experiments based on these data. The mean and standard deviation of muscle fiber CSA were used to determine the effect size between control and repair groups. A sample size of *N* = 6 per group (1:1 ratio between groups) would be required to achieve a power level > 0.8 with α = 0.05. Calculations were performed in G*Power (version 3; Dusseldorf, Germany) (Faul et al., 2007).

## Discussion

This study provides a detailed timeline of gross and histological changes in the rabbit supraspinatus model of chronic tear repair, which successfully replicates several key aspects of human RC repair. Acute worsening of muscle atrophy did occur at 1–2 weeks post-repair, confirming our first hypothesis. However, the accompanying peak in muscle degeneration at 2 weeks was not predicted. Our second hypothesis was supported by a lack of regeneration across all timepoints. Finally, our third hypothesis was disproven for atrophy and degeneration, and only partially true for fatty infiltration. Both fat accumulation and fibrosis were consistently increased after repair across all timepoints. Overall, these results mirror the clinical finding that repair induces a “second-hit” phenomenon with regard to muscle atrophy, while fat accumulation and fibrosis are persistent. A unique contribution of the present study was to verify the reproducibility of this model, with different surgeons in separate years generating similar results. These data can inform future translational studies that seek to use an animal model which accurately and reliably recapitulates the clinical course of RC repair. It also provides a series of reference points for time-specific comparisons.

Changes in muscle atrophy and degeneration appeared more time-limited than the changes in fat. Muscle mass decreased 26 ± 6% at 2 weeks post-repair, correlating with the largest percentage difference (54 ± 7%) in muscle fiber CSA. By 8 weeks post-repair (16 weeks post-tenotomy), mass was not statistically different and fiber CSA difference improved to 35 ± 6%. For comparison, our previous study found a 26.5% reduction in muscle fiber CSA at 16 weeks post-tenotomy without repair (Vargas-Vila et al., 2021). However, that study used contralateral sham surgery as the control, so it is unknown whether the fiber atrophy would have been the same or greater compared to uninjured tissue. In sheep, a similar pattern of muscle atrophy is seen after repair. The infraspinatus area on CT was around 28% less than the contralateral side at 40 weeks post-tenotomy, when repair was performed (Gerber et al., 2004). It decreased significantly to roughly 36% at 6 weeks after repair, partly recovered at 12 weeks, and remained approximately 22% smaller than the other side at 35 weeks after repair (Gerber et al., 2004). The current study provides evidence that this short-term spike in muscle atrophy after repair can be replicated in a smaller scale animal model.

Evidence of acute worsening of muscle atrophy after repair in this rabbit model is notable as prior work has shown conflicting results when the rabbits are sacrificed at later timepoints. When Fabis et al. (2016) performed repair at 12 weeks and sacrifice 24 weeks later, they found that atrophy had reversed, because there was no difference in type I or type II muscle fiber diameter between the repaired muscles and unoperated controls. However, reversal of atrophy has not been observed in other studies that examined muscle tissue and muscle belly volume, which showed decreases on the repair side compared to control at similar 12 and 24 week timepoints (Matsumoto et al., 2002; Uhthoff et al., 2003, 2014a). The present study offers a clearer understanding of changes in muscle atrophy across different timepoints at both the whole muscle and fiber level. A similar pattern has previously been seen when the tenotomy and repair are combined in the same event. After decreasing significantly at 1 and 2 weeks after immediate repair, muscle mass recovers to control level by 6 weeks, but fat continues to accumulate progressively over time (Uhthoff et al., 2014b).

Like muscle atrophy, degeneration also peaked at 2 weeks post-repair, with signs of active degeneration found in roughly 8 ± 5% of grid areas. Qualitatively, Gerber (Gerber et al., 2004) observed similar signs in sheep at 6 weeks post-repair, reporting “additional evidence of fiber degeneration with a lack of homogeneity of diameter and distribution. Some fibers were degenerated, and others showed signs, such as central nuclei, of reorganization.” This present study in rabbits was able to quantify not only degeneration, but the lack of significant regeneration. The slight rise in regeneration at 2 weeks appeared similar to the contralateral side, and neither treatment nor time had a significant effect. Similar lack of regeneration has been shown after chronic tenotomy in rabbits (Valencia et al., 2018; Vargas-Vila et al., 2021), but to our knowledge this is the first report after repair. In humans with end-stage RC disease undergoing shoulder arthroplasty, the percentage of centrally nucleated fibers was as high as 11.3%, but the prevalence of degenerated fibers was also 90% (Gibbons et al., 2017).

Previous rabbit studies have suggested that timing of “early” versus “delayed” tendon repair does not prevent significant fat accumulation (Matsumoto et al., 2002; Uhthoff et al., 2003, 2014a). Those authors postulated that fat accumulation could be induced by the trauma of a second surgery, or by the temporary reduction in activity of rabbits post-operatively. In the current study, fat accumulation was significantly increased in the repair shoulders throughout the study period, with no significant changes over time. Similarly, in sheep, a slight increase in fatty infiltration between the time of repair and 6 weeks after did not reach significance, nor did the slight decrease between 6 weeks and 12 weeks after repair (Gerber et al., 2004). Like in this study, the differences between the repair side and control were significant across all post-repair timepoints (Gerber et al., 2004). Again, this demonstrates that the rabbit model is able to accurately replicate the injury patterns seen in larger-scale animal models of RC disease. It also provides clarity on the relative effect of repair on muscle atrophy versus fat accumulation, with the latter not demonstrating significant worsening after repair. In terms of fibrosis, both OHP assay and trichrome showed that repaired tissue is significantly more fibrotic than uninjured control. Elevated fibrosis is compatible with the measured increase in active muscle degeneration, as well as inflammatory changes qualitatively observed on H&E-stained slides. By characterizing acute tissue responses to chronic tear repair, these results further our understanding of potential mechanisms to target with future therapies and provide time-specific benchmarks against which to measure their effects. For example, an intervention targeting muscle degeneration is likely best applied within the first 2 weeks after repair.

Based on the data in this study, *N* = 6 rabbits per group should be used for future experiments to achieve a power level > 0.8 with α = 0.05. Duplication of the experiments demonstrated strong reliability of this model even with different surgeons in non-overlapping years, though some of the histology and other analyses were carried out by the same personnel. There were minimal differences in most experimental measurements, with only muscle regeneration (% central nuclei) showing a significant difference in the ANOVA. This likely reflects a noisier measurement due to the low incidence of muscle regeneration (less than 4%) seen across all specimens. Another possible explanation is the use of the grid-based grading system, as fat percentage determined by H&E-stained grid areas also differed in the same 2- and 4-week control shoulders. In contrast, fat accumulation by ORO staining showed no changes between rounds of experimentation and may be a more reliable method of quantification. This information may help guide future researchers in the selection of tools for measuring tissue changes in the rabbit RC model.

There are a few limitations to consider when interpreting the results of this study. The control arm consisted of unoperated contralateral shoulders, rather than tenotomy only, sham surgery, or a separate group of animals. However, the results do demonstrate consistency of the model, and a previous set of tenotomy + sham surgery experiments has been performed in our lab as a historical control (Vargas-Vila et al., 2021). Secondly, all repairs were performed at a single timepoint after tenotomy (8 weeks), whereas other authors have examined differential results from repairs performed immediately or at various times. Conversely, a strength of this study was the use of multiple timepoints for sacrifice to show the changes in tissue over time.

## Conclusion

Repair of a chronically torn supraspinatus in rabbits leads to a short-term increase in muscle degeneration and atrophy that peaks at 2 weeks post-repair. Fibrosis and fatty infiltration did not change as a function of time in the period studied here, but both remained significantly elevated compared to the uninjured side. Finally, the two rounds of experimentation yielded similar results overall, demonstrating the reproducibility of this pre-clinical RC model.

## Data Availability Statement

The raw data supporting the conclusions of this article will be made available by the authors, without undue reservation.

## Ethics Statement

The animal study was reviewed and approved by the UC San Diego Institutional Animal Care and Use Committee.

## Author Contributions

MG, JL, AS, and SW were responsible for the conception and design of the study. LV-B, MG, SD, SR, IW, ME, DF, JL, SH, and AS contributed to the collection and analysis of data. IW and LV-B were responsible for the design and drafting of the manuscript. All authors revised the manuscript and gave final approval.

## Conflict of Interest

The authors declare that the research was conducted in the absence of any commercial or financial relationships that could be construed as a potential conflict of interest.

## Publisher’s Note

All claims expressed in this article are solely those of the authors and do not necessarily represent those of their affiliated organizations, or those of the publisher, the editors and the reviewers. Any product that may be evaluated in this article, or claim that may be made by its manufacturer, is not guaranteed or endorsed by the publisher.

## References

[B1] Barton E. R. Gimbel J. A. Williams G. R. Soslowsky L. J. (2005). Rat supraspinatus muscle atrophy after tendon detachment. *J. Orthop. Res.* 23 259–265. 10.1016/j.orthres.2004.08.018 15734235

[B2] Cho N. S. Rhee Y. G. (2009). The factors affecting the clinical outcome and integrity of arthroscopically repaired rotator cuff tears of the shoulder. *Clin. Orthop. Surg.* 1 96–104. 10.4055/cios.2009.1.2.96 19885061 PMC2766755

[B3] Chung S. W. Song B. W. Kim Y. H. Park K. U. Oh J. H. (2013). Effect of platelet-rich plasma and porcine dermal collagen graft augmentation for rotator cuff healing in a rabbit model. *Am. J. Sports Med.* 41 2909–2918. 10.1177/0363546513503810 24047553

[B4] Collin P. Treseder T. Ladermann A. Benkalfate T. Mourtada R. Courage O. (2014). Neuropathy of the suprascapular nerve and massive rotator cuff tears: a prospective electromyographic study. *J. Shoulder Elbow Surg.* 23 28–34. 10.1016/j.jse.2013.07.039 24090983

[B5] Derwin K. A. Baker A. R. Iannotti J. P. McCarron J. A. (2010). Preclinical models for translating regenerative medicine therapies for rotator cuff repair. *Tissue Eng. Part B Rev.* 16 21–30. 10.1089/ten.TEB.2009.0209 19663651 PMC2817667

[B6] Edwards C. A. O’Brien W. D. Jr. (1980). Modified assay for determination of hydroxyproline in a tissue hydrolyzate. *Clin. Chim. Acta* 104 161–167. 10.1016/0009-8981(80)90192-8 7389130

[B7] Fabis J. Danilewicz M. Zwierzchowski J. T. Niedzielski K. (2016). Atrophy of type I and II muscle fibers is reversible in the case of grade >2 fatty degeneration of the supraspinatus muscle: an experimental study in rabbits. *J. Shoulder Elbow Surg.* 25 487–492. 10.1016/j.jse.2015.08.034 26549862

[B8] Fabis J. Kordek P. Bogucki A. Mazanowska-Gajdowicz J. (2000). Function of the rabbit supraspinatus muscle after large detachment of its tendon: 6-week, 3-month, and 6-month observation. *J. Shoulder Elbow Surg.* 9 211–216. 10888165

[B9] Fabis J. Kordek P. Bogucki A. Synder M. Kolczynska H. (1998). Function of the rabbit supraspinatus muscle after detachment of its tendon from the greater tubercle: observations up to 6 months. *Acta Orthop. Scand.* 69 570–574. 10.3109/17453679808999257 9930099

[B10] Faul F. Erdfelder E. Lang A. G. Buchner A. (2007). G*Power 3: a flexible statistical power analysis program for the social, behavioral, and biomedical sciences. *Behav. Res. Methods* 39 175–191. 10.3758/bf03193146 17695343

[B11] Galatz L. M. Ball C. M. Teefey S. A. Middleton W. D. Yamaguchi K. (2004). The outcome and repair integrity of completely arthroscopically repaired large and massive rotator cuff tears. *J. Bone Joint Surg. Am.* 86 219–224. 10.2106/00004623-200402000-00002 14960664

[B12] Gerber C. Meyer D. C. Schneeberger A. G. Hoppeler H. von Rechenberg B. (2004). Effect of tendon release and delayed repair on the structure of the muscles of the rotator cuff: an experimental study in sheep. *J. Bone Joint Surg. Am.* 86 1973–1982. 10.2106/00004623-200409000-00016 15342760

[B13] Gibbons M. C. Singh A. Anakwenze O. Cheng T. Pomerantz M. Schenk S. (2017). Histological evidence of muscle degeneration in advanced human rotator cuff disease. *J. Bone Joint Surg. Am.* 99 190–199. 10.2106/JBJS.16.00335 28145949 PMC5395080

[B14] Gladstone J. N. Bishop J. Y. Lo I. K. Flatow E. L. (2007). Fatty infiltration and atrophy of the rotator cuff do not improve after rotator cuff repair and correlate with poor functional outcome. *Am. J. Sports Med.* 35 719–728. 10.1177/0363546506297539 17337727

[B15] Honda H. Gotoh M. Kanazawa T. Ohzono H. Nakamura H. Ohta K. (2017). Hyaluronic acid accelerates tendon-to-bone healing after rotator cuff repair. *Am. J. Sports Med.* 45 3322–3330. 10.1177/0363546517720199 28872895

[B16] Kwon J. Kim S. H. Lee Y. H. Kim T. I. Oh J. H. (2019). The rotator cuff healing index: a new scoring system to predict rotator cuff healing after surgical repair. *Am. J. Sports Med.* 47 173–180. 10.1177/0363546518810763 30485753

[B17] Kwon J. Kim Y. H. Rhee S. M. Kim T. I. Lee J. Jeon S. (2018). Effects of allogenic dermal fibroblasts on rotator cuff healing in a rabbit model of chronic tear. *Am. J. Sports Med.* 46 1901–1908. 10.1177/0363546518770428 29746144

[B18] Li X. Shen P. Su W. Zhao S. Zhao J. (2018). Into-Tunnel repair versus onto-surface repair for rotator cuff tears in a rabbit model. *Am. J. Sports Med.* 46 1711–1719. 10.1177/0363546518764685 29620913

[B19] Liu X. Laron D. Natsuhara K. Manzano G. Kim H. T. Feeley B. T. (2012). A mouse model of massive rotator cuff tears. *J. Bone Joint Surg. Am.* 94:e41. 10.2106/JBJS.K.00620 22488625

[B20] Liu X. Manzano G. Kim H. T. Feeley B. T. (2011). A rat model of massive rotator cuff tears. *J. Orthop. Res.* 29 588–595. 20949443 10.1002/jor.21266

[B21] Matsumoto F. Uhthoff H. K. Trudel G. Loehr J. F. (2002). Delayed tendon reattachment does not reverse atrophy and fat accumulation of the supraspinatus–an experimental study in rabbits. *J. Orthop. Res.* 20 357–363. 10.1016/S0736-0266(01)00093-6 11918317

[B22] McElvany M. D. McGoldrick E. Gee A. O. Neradilek M. B. Matsen F. A. III (2015). Rotator cuff repair: published evidence on factors associated with repair integrity and clinical outcome. *Am. J. Sports Med.* 43 491–500. 10.1177/0363546514529644 24753240

[B23] Ozbaydar M. Elhassan B. Esenyel C. Atalar A. Bozdag E. Sunbuloglu E. (2008). A comparison of single-versus double-row suture anchor techniques in a simulated repair of the rotator cuff: an experimental study in rabbits. *J. Bone Joint Surg. Br.* 90 1386–1391. 10.1302/0301-620X.90B10.20862 18827253

[B24] Park J. S. Park H. J. Kim S. H. Oh J. H. (2015). Prognostic factors affecting rotator cuff healing after arthroscopic repair in small to medium-sized tears. *Am. J. Sports Med.* 43 2386–2392. 10.1177/0363546515594449 26286879

[B25] Phillips D. I. Caddy S. Ilic V. Fielding B. A. Frayn K. N. Borthwick A. C. (1996). Intramuscular triglyceride and muscle insulin sensitivity: evidence for a relationship in nondiabetic subjects. *Metabolism* 45 947–950. 10.1016/s0026-0495(96)90260-7 8769349

[B26] Rowshan K. Hadley S. Pham K. Caiozzo V. Lee T. Q. Gupta R. (2010). Development of fatty atrophy after neurologic and rotator cuff injuries in an animal model of rotator cuff pathology. *J. Bone Joint Surg. Am.* 92 2270–2278. 10.2106/JBJS.I.00812 20926720 PMC2945930

[B27] Shi L. L. Boykin R. E. Lin A. Warner J. J. (2014). Association of suprascapular neuropathy with rotator cuff tendon tears and fatty degeneration. *J. Shoulder Elbow Surg.* 23 339–346. 10.1016/j.jse.2013.06.011 24054975

[B28] Su W. Li X. Zhao S. Shen P. Dong S. Jiang J. (2018). Native enthesis preservation versus removal in rotator cuff repair in a rabbit model. *Arthroscopy* 34 2054–2062. 10.1016/j.arthro.2018.03.005 29789248

[B29] Sun Y. Kwak J. M. Kholinne E. Zhou Y. Tan J. Koh K. H. (2020). Small subchondral drill holes improve marrow stimulation of rotator cuff repair in a rabbit model of chronic rotator cuff tear. *Am. J. Sports Med.* 48 706–714. 10.1177/0363546519896350 31928410

[B30] Talmadge R. J. Roy R. R. (1993). Electrophoretic separation of rat skeletal muscle myosin heavy-chain isoforms. *J. Appl. Physiol. (1985)* 75 2337–2340. 10.1152/jappl.1993.75.5.2337 8307894

[B31] Trudel G. Uhthoff H. K. Wong K. Dupuis J. Laneuville O. (2019). Adipocyte hyperplasia: the primary mechanism of supraspinatus intramuscular fat accumulation after a complete rotator cuff tendon tear: a study in the rabbit. *Adipocyte* 8 144–153. 10.1080/21623945.2019.1609201 31033395 PMC6768259

[B32] Uhthoff H. K. Coletta E. Trudel G. (2014a). Effect of timing of surgical SSP tendon repair on muscle alterations. *J. Orthop. Res.* 32 1430–1435. 10.1002/jor.22692 25070492

[B33] Uhthoff H. K. Coletta E. Trudel G. (2014b). Intramuscular fat accumulation and muscle atrophy in the absence of muscle retraction. *Bone Joint Res.* 3 117–122. 10.1302/2046-3758.34.2000275 24743593 PMC4036302

[B34] Uhthoff H. K. Matsumoto F. Trudel G. Himori K. (2003). Early reattachment does not reverse atrophy and fat accumulation of the supraspinatus–an experimental study in rabbits. *J. Orthop. Res.* 21 386–392. 10.1016/S0736-0266(02)00208-5 12706009

[B35] Valencia A. P. Lai J. K. Iyer S. R. Mistretta K. L. Spangenburg E. E. Davis D. L. (2018). Fatty infiltration is a prognostic marker of muscle function after rotator cuff tear. *Am. J. Sports Med.* 46 2161–2169. 10.1177/0363546518769267 29750541 PMC6397750

[B36] Vargas-Vila M. A. Gibbons M. C. Wu I. T. Esparza M. C. Kato K. Johnson S. D. (2021). Progression of muscle loss and fat accumulation in a rabbit model of rotator cuff tear. *J. Orthop. Res.* 1–10. 10.1002/jor.25160 34392563 PMC8844305

[B37] Yoon J. P. Lee C. H. Jung J. W. Lee H. J. Lee Y. S. Kim J. Y. (2018). Sustained delivery of transforming growth factor beta1 by use of absorbable alginate scaffold enhances rotator cuff healing in a rabbit model. *Am. J. Sports Med.* 46 1441–1450. 10.1177/0363546518757759 29543511

[B38] Zhou X. Moore B. B. (2017). Lung section staining and microscopy. *Bio Protoc.* 7:e2286. 10.21769/BioProtoc.2286 29170747 PMC5697772

