# Supplementary material for: The “Second Hit” of Repair in a Rabbit Model of Chronic Rotator Cuff Tear
Source: Front Physiol. 2022 Mar 8;13:801829. doi: 10.3389/fphys.2022.801829 (PMC8958027; doi:10.3389/fphys.2022.801829)
Supplement: Supplementary file 1 [file Data_Sheet_1.PDF]

Supplementary Material

1 Supplementary Figures

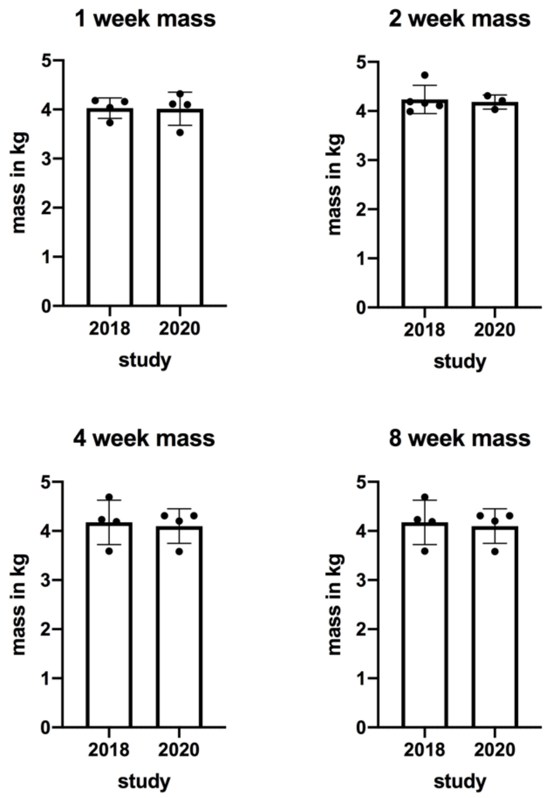

Supplemental Figure 1. Comparison of whole animal mass at sacrifice, *i.e.* 8 weeks of tenotomy +1/2/4/8 weeks after repair, 2018 versus 2020 data. Mean ± SD shown; horizontal bar denotes $p < 0.05$.

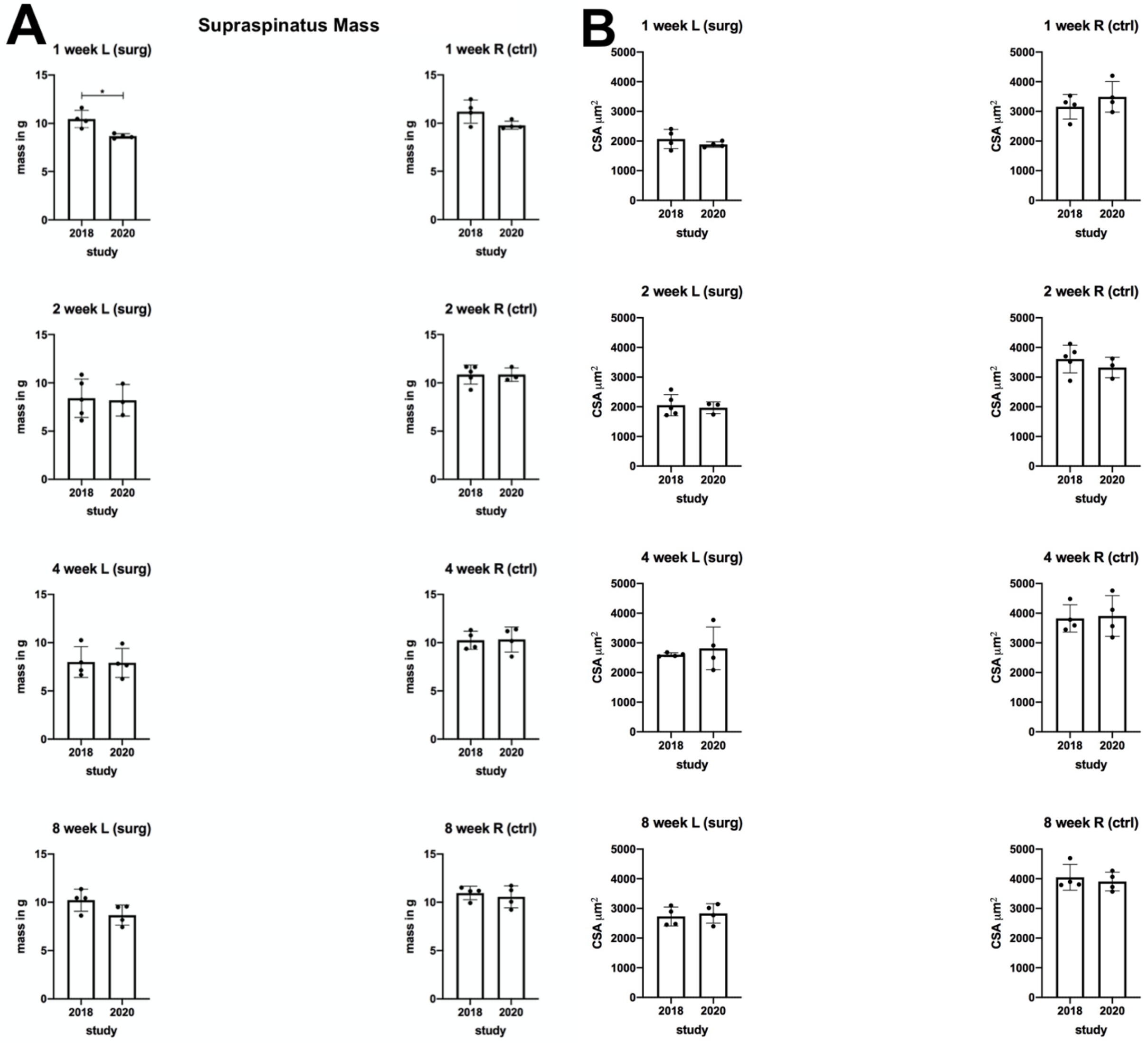

Supplemental Figure 2. Comparison of (A) supraspinatus muscle mass and (B) muscle fiber cross-sectional area (CSA) in 2018 versus 2020 data. Mean ± SD shown; horizontal bar denotes $p < 0.05$.

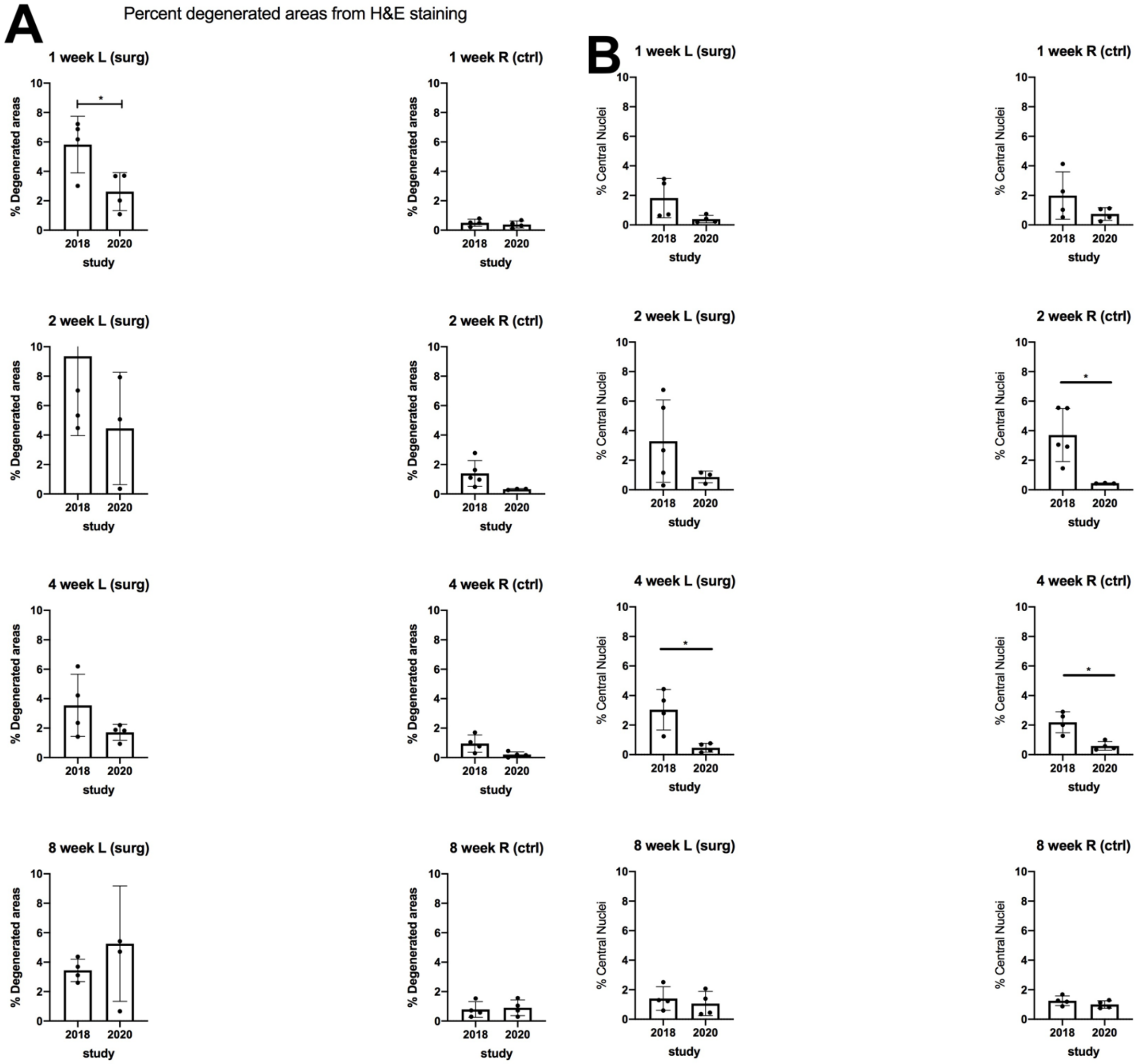

Supplemental Figure 3. Comparison of 2018 versus 2020 data on (A) percentage of H&E grid areas demonstrating signs of muscle degeneration and (B) percentage of muscle fibers with centralized nuclei. Mean ± SD shown; horizontal bar denotes $p < 0.05$.

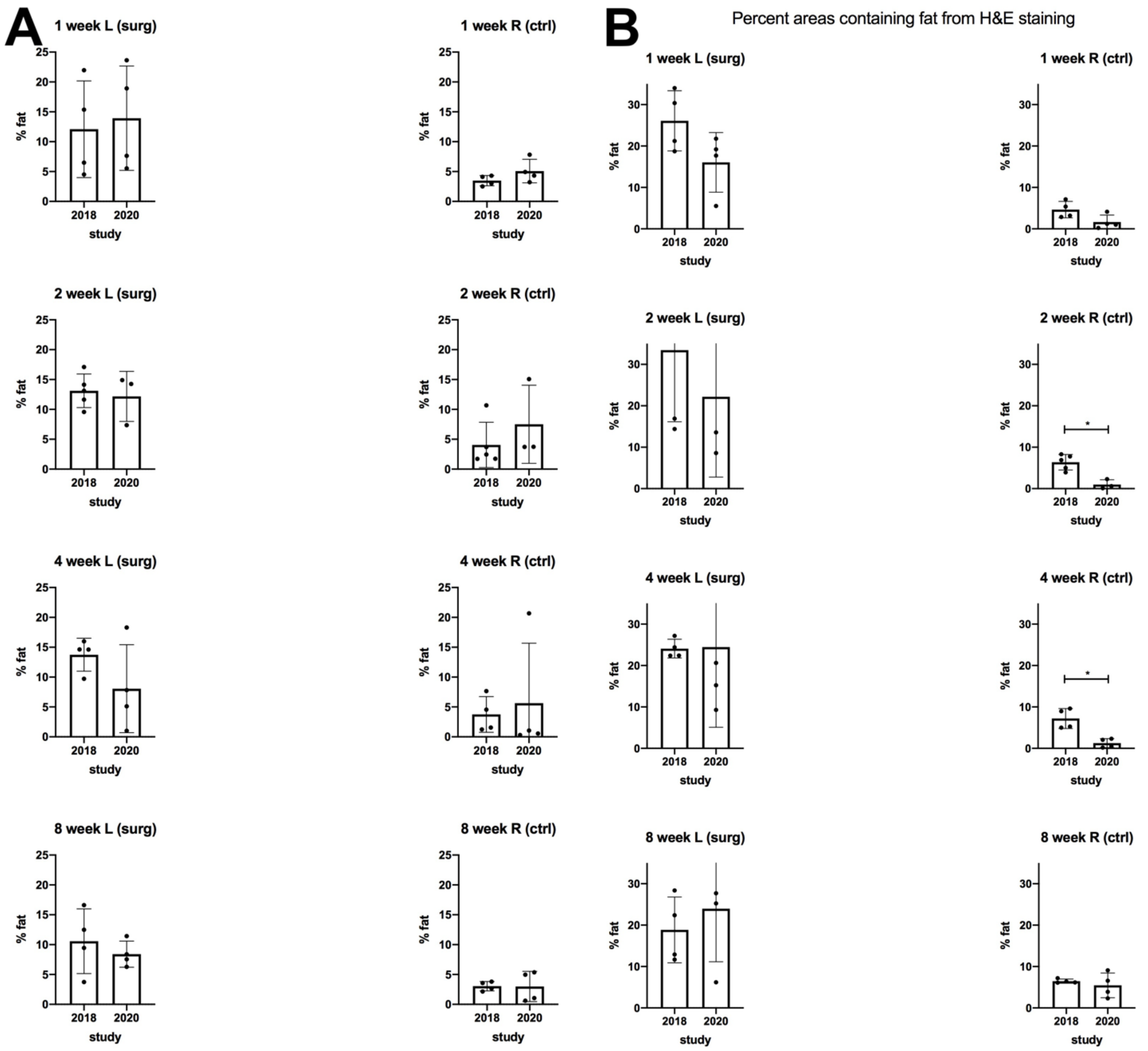

Supplemental Figure 4. Comparison of percentage of cross-sectional area (CSA) occupied by fat based on (A) semi-automated quantification of red after Oil Red O staining, or (B) grading of grid areas as positive or negative for fat. Mean ± SD shown; horizontal bar denotes $p < 0.05$.

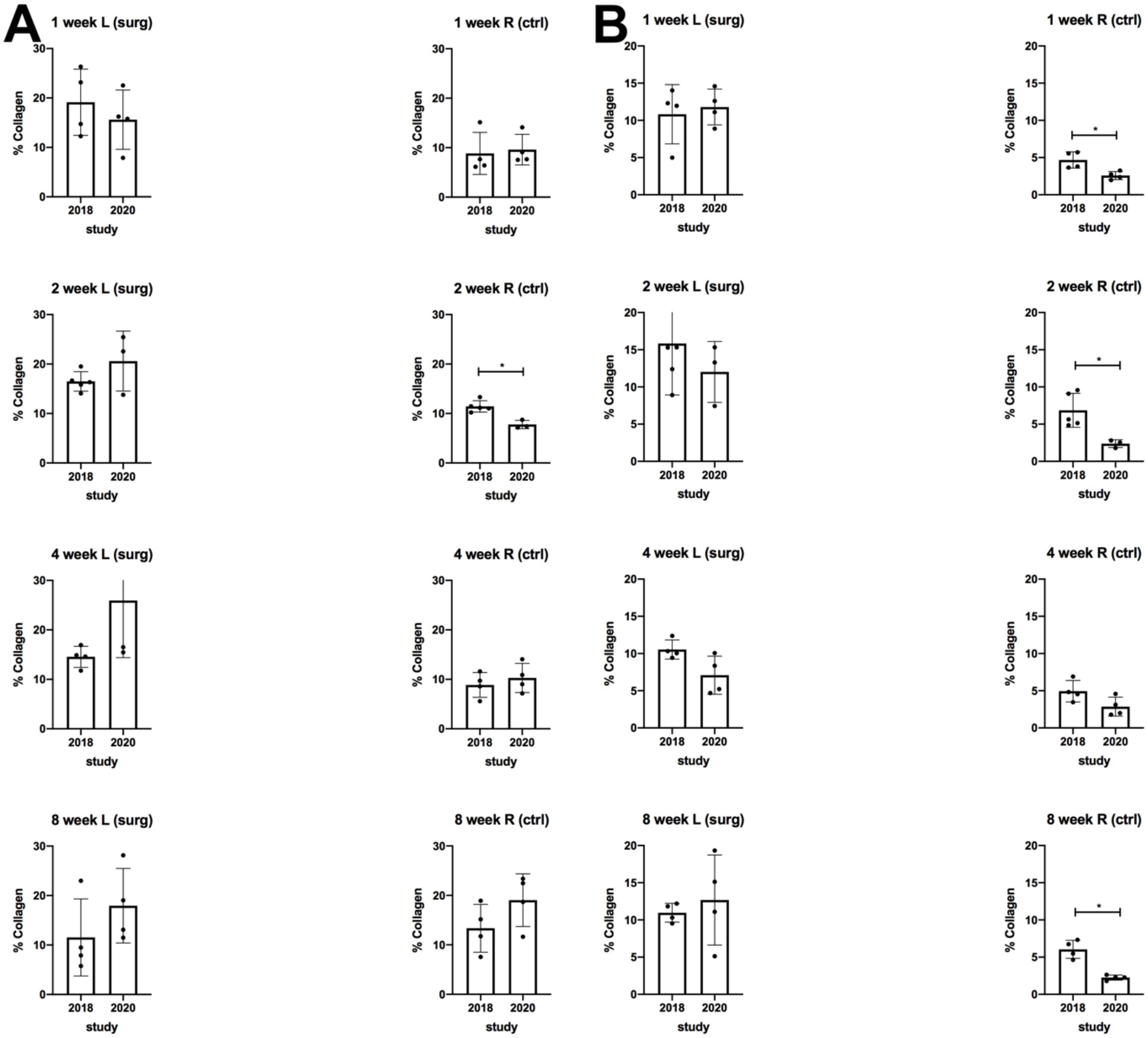

Supplemental Figure 5. Comparison of supraspinatus collagen content using (A) hydroxyproline assay and (B) trichrome staining in rabbits from 2018 versus 2020 data. Mean ± SD shown; horizontal bar denotes $p < 0.05$.
